# TIMELY MUNICIPALITY REHABILITATION AFTER HOSPITALISATION REDUCES READMISSION AND EARLY MORTALITY

**DOI:** 10.2340/jrm-cc.v7.40636

**Published:** 2024-09-05

**Authors:** Søren BIE BOGH, Sören MÖLLER, Mette BIRK-OLSEN, Lars MORSØ

**Affiliations:** 1 Research Unit OPEN, Department of Clinical Research, University of Southern Denmark, Odense, Denmark; 2 Open Patient Data Explorative Network, Odense University Hospital, Region of Southern Denmark, Odense, Denmark; 3 The Department of the Elderly and Disabled, Odense, Denmark

**Keywords:** rehabilitation, hospitalisation, readmission, municipality, mortality

## Abstract

**Objective:**

Firstly, the study explores the association between timely initiation of rehabilitation and 90-day and 365-day all-cause acute readmission and secondly, 90-day and 365-day all-cause mortality in a cohort of Odense Municipality residents.

**Methods:**

The registry-based observational cohort study investigates acute contacts at Odense University Hospital from 2015 to 2020. Descriptive statistics, Cox regression and cumulative incidence rates were used for analysis.

**Subjects:**

The study utilizes initiated rehabilitation referrals within 60 days from Odense Municipality residents.

**Results:**

In total, 7,377 rehabilitation plans were initiated, including 5051 (68.5%) within the legal timeframe. Overall, timely initiation of rehabilitation within the legal timeframe was associated with a significantly reduced risk of 90-day all-cause acute readmission (Adjusted HR 0.82, 95% CI 0.74–0.90).

In the adjusted analysis, timely initiation was also significantly associated with reduced risk in 365-day all-cause acute readmission (HR 0.90, 95% CI 0.83–0.97). Each week of delay in initiation of rehabilitation was associated with an increased risk of readmission (HR 1.05, 95% CI 1.02–1.07). Further, timely initiation of rehabilitation was associated with a significant reduction in the risk of 365-day all-cause mortality (HR 0.74, 95% CI 0.61–0.89).

**Conclusion:**

Timely initiation of rehabilitation within the legal timeframe of 7 or 14 days was associated with significantly reduced risk of 90-day and 365-day all-cause acute readmission. Timely initiation of rehabilitation was also associated with significant reduction in the risk of 365-day all-cause mortality.

## Lay Abstract

Most patients can benefit from rehabilitation after hospital admission. Early rehabilitation has shown to be useful for specific patient groups. This study explores the association between timely rehabilitation and readmission and early mortality in a cohort of Odense Municipality residents.

Findings show that timely initiation of rehabilitation is associated with fewer acute readmissions. For each week the rehabilitation is delayed the risk of readmission increases. Furthermore, timely initiation of rehabilitation was significantly associated with a reduction of all-cause mortality within 365 days.

Musculoskeletal, mental, neurological and respiratory diseases are future contributors to 310 million years of life lived with disability (1). This, combined with demographic development, results in an increased number of elderly, bringing healthcare services under pressure (2). Rehabilitation is recommended in order to face the huge global burden of diseases and as part of the solution to future demographic challenges (3, 4). It is estimated that 2.4 billion individuals have conditions that could benefit from rehabilitation and that one in three needs rehabilitation at some point in life (4).

Deconditioning after acute hospital admission resulting in reduced physical performance is evident (5, 6). The hospital associated deconditioning increases the risk of mortality, readmission and institutionalisation the following year (7, 8). Therefore, it is crucial to face these negative consequences of hospital admission.

In Denmark, patients who experience a decline in physical ability after hospital admission are entitled to be prescribed a rehabilitation plan for municipality rehabilitation that should be initiated within 7 days (earlier 14 days) according to Danish law.

Initiation of timely rehabilitation after hospital admission poses some challenges. This is due to coordination across care settings (9) or due to patient related issues (10). In a prior study, we found that increased age and nursing home residency increased the chance of timely rehabilitation initiation, while nursing care and high comorbidity significantly decreased the chance of timely initiation (unpublished).

Studies have shown that early rehabilitation after discharge increases physical activity and quality of life (4, 11) and have shown that early rehabilitation plays a significant role in preventing readmission and mortality in various populations of chronic illnesses, disabilities and patients recovering from acute medical events such as stroke (4, 12). These studies investigate study populations with specific diagnoses. Studies using data from an entire municipality population have not been conducted.

This register-based observational cohort study uses all acute contacts in a municipality population to explore (1) associations of timely initiated rehabilitation according to Danish law and 90-day and 1-year readmission, (2) associations of timely initiated rehabilitation according to Danish law and 90-day and 1-year mortality.

## METHODS AND RESULTS

### Design and participants

The study included adult (≥ 18 years) acute patients treated at Odense University Hospital (OUH) between 2015 and 2020. During that period, 225,653 Odense Municipality residents were discharged from the hospital, and around 5% (10.327) were discharged with a rehabilitation plan. Only participants whose rehabilitation plan was initiated within 60 days of hospital discharge were included. Sixty-day initiation was chosen to ensure the rehabilitation plan was initiated within a reasonable time after discharge.

### Analysis

We conducted Cox proportional hazard analyses to assess the relationship between timely initiation of rehabilitation and four outcomes: 90-day and 365-day all-cause acute readmission and 90-day and 365-day all-cause mortality. Rehabilitation was defined as being timely when initiated within a legal timeframe of 14 days before and 7 days after June 22, 2018, at which timepoint the Danish regulation changed. In addition, we examined the association between delayed initiation per week and readmission. We did not analyse the association between weekly delayed initiation and mortality due to a low number of events. Analyses were performed unadjusted and adjusted for covariates: sex, age, comorbidities (Charlson index score (13)), education level, civil status, ethnicity, residence in a nursing home, and allocated nursing and domestic care. To mitigate immortal time bias, the risk time began at the date of initiation or at the end of the timely rehabilitation initiation period, whichever occurred first. In the readmission analyses, death occurrences were treated as censoring events, and conversely, readmissions were considered censoring events in mortality analyses.

Statistical analyses and data management were conducted using Stata 18 (Stata Corp LP, College Station, TX), with a significance level set at 5%.

### Results

In total, 7377 rehabilitation plans were initiated, of which 5051 (68.5%) were initiated within the legal timeframe. Patients with timely initiation were more often female, more frequently above 80 years, had higher comorbidity and received less nursing care. Please see Table I for more details on patient characteristics and the Table SI for discharge diagnosis information.

**Table I:** Comparison of patient characteristics based on timely versus non-timely rehabilitation

	Non-timely rehabilitation		Timely rehabilitation		*p*-value

	*N*=2326		*N*=5051		

	*n*	%	*n*	%	
Sex					
Female	1,253	53.9	2845	56.3	0.076
Male	1,073	46.1	2206	43.7	
Age groups					
18–49 years	192	8.3	198	3.9	<0.001
50–64 years	320	13.8	565	11.2	
65–79 years	823	35.4	1,776	35.2	
80+ years	991	42.6	2,512	49.7	
Civil status					
Single	1,484	63.8	3276	64.9	0.32
Partnered	842	36.2	1,,775	35.1	
Ethnicity					
Foreign	156	6.7	268	5.3	0.52
Danish	2,170	93.3	4,783	94.7	
Education level					
None/low	1,097	47.2	2,509	49.7	0.3
Medium	813	35.0	1,773	35.1	
High	416	17.9	769	15.2	
Comorbidity^a^					
Low	408	17.5	626	12.4	<0.001
Medium	616	26.5	1,101	21.8	
High	1,302	56.0	3,324	65.8	
Domestic help					
No	1,178	50.6	2,486	49.2	<0.001
Yes	1,148	49.4	2,565	50.8	
Nursing care					
No	1,472	63.6	3,471	68.7	<0.001
Yes	854	36.7	1,580	31.3	
Nursing home					
No	2,244	96.5	4,753	94.1	0.089
Yes	82	3.5	298	5.9	

a Charlson comorbidity index: 0=low, 1–2=moderate, >2=high.

Timely initiation of the rehabilitation plan within the legal timeframe was associated with a significantly reduced risk of 90-day all-cause acute readmission (Unadjusted HR 0.87, 95% CI 0.78–0.96; Adjusted HR 0.82, 95% CI 0.74–0.90). Each weekly delay in initiation was associated with an increased risk of readmission (Unadjusted HR 1.07, 95% CI 1.04–1.11; Adjusted HR 1.07, 95% CI 1.04–1.10).

**Fig. 1:**
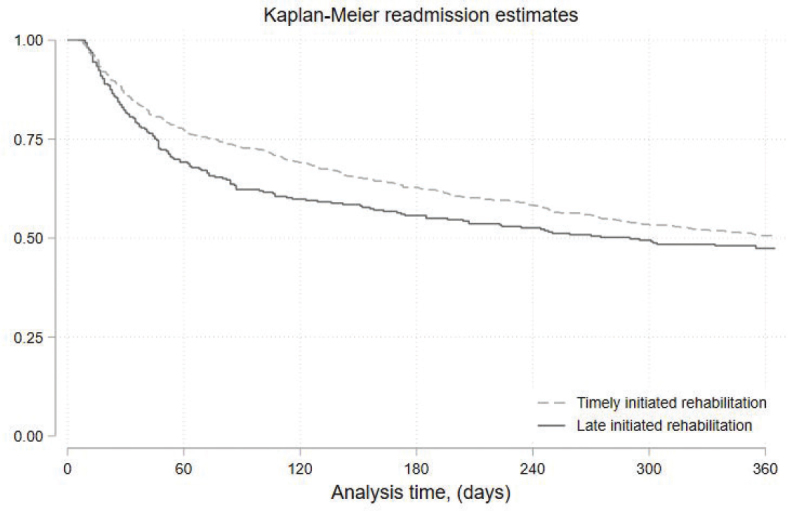
Kaplan-Meier survival curves. Illustrating readmission status based on timely (dotted line) versus late (solid line) initiation of the prescribed rehabilitation plan.

Regarding 365-day all-cause acute readmission, timely initiating within the legal timeframe showed a non-significant trend towards reduced risk in the unadjusted analysis (HR 0.95, 95% CI 0.88–1.03), which became significant in the adjusted analysis (HR 0.90, 95% CI 0.83–0.97). The reduced risk is also displayed in Fig. 1. Each weekly delay in initiation was associated with increased risk of readmission (Unadjusted HR 1.05, 95% CI 1.02–1.07; Adjusted HR 1.05, 95% CI 1.02–1.07).

Timely initiation of rehabilitation showed no significant association with 90-day all-cause mortality (Unadjusted HR 0.82, 95% CI 0.63–1.08; Adjusted HR 0.82, 95% CI 0.62–1.08). It was associated with a significant reduction in risk of 365-day all-cause mortality in both unadjusted (HR 0.75, 95% CI 0.64–0.88) and adjusted analyses (HR 0.74, 95% CI 0.61–0.89) (Table II).

**Table II:** Unadjusted and adjusted association of initiating a rehabilitation plan within the legal timeframe and weekly delayed initiation on patient outcomes

	Unadjusted			Adjusted†		

	HR	95% CI	*p*-value	HR	95% CI	*p*-value
**90-day all-cause acute readmission**						
Effect of initiation within the legal timeframe	0.87	0.78–0.96	0.005	0.82	0.74–0.90	<0.001
Effect of weekly delayed initiation	1.07	1.04–1.11	<0.001	1.07	1.04–1.10	<0.001
**365-day all-cause acute readmission**						
Effect of initiation within the legal timeframe	0.95	0.88–1.03	0.209	0.90	0.83–0.97	0.006
Effect of weekly delayed initiation	1.05	1.02–1.07	<0.001	1.05	1.02–1.07	<0.001
**90-day all-cause mortality**						
Effect of initiation within the legal timeframe	0.82	0.63–1.08	0.164	0.82	0.62–1.08	0.162
**365-day all-cause mortality**						
Effect of initiation within the legal timeframe	0.75	0.64–0.88	0.001	0.74	0.61–0.89	0.002

The outcomes assessed include 90-day all-cause acute readmission, 90-day all-cause mortality, and 365-day all-cause mortality.

HR: Hazard ratio; CI: Confidence interval.

† Adjusted for sex, age, comorbidity, education level, civil status, ethnicity, living at a nursing home, allocated nursing care and domestic care.

## DISCUSSION

The study showed that timely initiation of the rehabilitation plan within the legal timeframe was associated with a significantly reduced risk of 90-day and 365-day all-cause acute readmission. For each week of delay in initiation of rehabilitation, the risk of readmission increased significantly and finally, timely initiation of rehabilitation was associated with a significant reduction in risk of 365-day all-cause mortality.

The findings of this study show the importance of early rehabilitation and align with findings in, e.g. studies on chronic obstructive pulmonary disease (14, 15). Most studies exploring rehabilitation’s effect are performed in specific cohorts or randomized controlled trials in selected diagnoses. Our register-based observational cohort study used all acute contacts across diagnoses in an entire municipality population. Still, timely rehabilitation seemed to be associated with reduced risk of readmission and reduction in 365-day mortality risk. Our findings show that timely rehabilitation was initiated not only by well-functioning younger patients but also by patients living in nursing homes, those aged over 80 and those dealing with comorbidities. Overall, our findings suggest that register based studies can inform the impact of timely rehabilitation.

In this study, we did not consider any patient barriers for initiation of rehabilitation. There are well-known barriers such as a lack of knowledge or exhaustion that make it challenging to initiate timely rehabilitation (16). With our findings in mind, clinicians might need to focus on providing more information to those in need. For those experiencing exhaustion, rehabilitation programmes should be promptly adapted and initiated (e.g. by applying home rehabilitation).

There are, of course, some limitations to this study. We have neither included any information on the content of rehabilitation nor the number of times patients who attended rehabilitation. There are some indications in our material showing that once rehabilitation is initiated – timely or not, patients are quite adherent. Due to a lack of attendance information, we are unable to qualify for dose-response relationships or contents of the rehabilitation. Therefore, we cannot answer whether the association of rehabilitation with reduced readmission risk and reduced 365-day mortality is due to increased physical ability or if social and psychological factors contribute to the association. Attending rehabilitation requires general mobilisation, which might account for some effects.

We have been censoring readmissions within the legal seven or 14-day initiation period. We know there have been readmissions within this timeframe and those who are re-admitted promptly are probably the most fragile. Therefore, the most fragile patients might have been censored even before the very initiation of the rehabilitation.

The legal initiation time point of rehabilitation was changed in June 2018 from 14 to 7 days. Prior results have shown that about 50% initiate rehabilitation within these first 2 weeks. Even though results indicate that patients should receive early rehabilitation, there might be a lower limit for initiation of rehabilitation due to practical considerations (e.g. referral time, initiation of nursing or domestic care).

Overall, there seem to be associations between initiating rehabilitation within the legal requirement of 7 or 14 days and reduced readmission and reduced 365-day mortality. This indicates that healthcare organisations should remain focused on early rehabilitation. Furthermore, future studies might need to focus on additional outcomes beyond readmission and mortality, e.g. years lived with increased quality of life or health service measures and municipality expenses.

## Supplementary Material


