# Supplementary material for: TIMELY MUNICIPALITY REHABILITATION AFTER HOSPITALISATION REDUCES READMISSION AND EARLY MORTALITY
Source: J Rehabil Med Clin Commun. 2024 Sep 5;7:40636. doi: 10.2340/jrm-cc.v7.40636 (PMC11388109; doi:10.2340/jrm-cc.v7.40636)
Supplement: Supplementary file 1 [file JRMCC-7-40636-s1.pdf]

Supplementary material has been published as submitted. It has not been copyedited, or typeset by Journal of Rehabilitation Medicine – Clinical Communications

Table SI. Comparison of Discharge diagnosis (ICD10 Chapters) based on timely versus non-timely rehabilitation

	**Non-timely rehabilitation N=2,326**	**Timely rehabilitation N=5,051**
Infectious diseases	75 (3.2%)	179 (3.5%)
Neoplasms	41 (1.8%)	63 (1.2%)
Blood diseases	9 (0.4%)	30 (0.6%)
Endocrine diseases	128 (5.6%)	280 (5.5%)
Mental disorders	34 (1.5%)	57 (1.1%)
Nervous system diseases	20 (0.9%)	42 (0.8%)
Eye diseases	<5 (<0.2%)	<5 (<0.1%)
Ear diseases	<5 (<0.2%)	7 (0.1%)
Circulatory diseases	131 (5.6%)	293 (5.8%)
Respiratory diseases	233 (10%)	509 (10.1%)
Digestive diseases	43 (1.9%)	134 (2.7%)
Skin diseases	<5 (<0.2%)	15 (0.3%)
Musculoskeletal diseases	154 (6.6%)	178 (3.5%)
Genitourinary diseases	67 (2.9%)	156 (3.1%)
Female diseases	<5 (<0.2%)	<5 (<0.1%)
Perinatale diseases	<5 (<0.2%)	<5 (<0.1%)
Congenital malformatio	<5 (<0.2%)	<5 (<0.1%))
Symptoms	658 (28.3%)	1,283 (25.4%)
Lesions posining	609 (26.2%)	1,493 (29.6%)
Other factors	112 (4.8%)	326 (6.5%)

Data are presented as n (%).
